# A smart vision assistance system designed for the cybathlon 2024 vision assistance race to support individuals with visual impairments

**DOI:** 10.1186/s12984-026-02092-6

**Published:** 2026-07-21

**Authors:** Simon Senoner, Simon Winkler, Lukas Neururer, Leon Ochtendung, Julia Jackermaier, Marcel Naderer, Bernhard Tschulnigg, Bernhard Hollaus, Yeongmi Kim

**Affiliations:** Medical and Health Technologies, MCI University of Applied Sciences, Universitätsstrasse 15, 6020 Innsbruck, Tyrol Austria

**Keywords:** Cybathlon, Computer vision, Object classification, Object detection, Obstacle avoidance, Vision assistance

## Abstract

Individuals with visual impairments continue to face challenges in performing everyday tasks, despite ongoing advances in assistive technologies. In the context of the Cybathlon 2024 Vision Assistance Race, we developed and evaluated a smart vision assistance system combining lightweight wearable hardware, multimodal sensing, and computer vision algorithms. This article presents the technical development process of the system, including hardware design, sensor integration, feedback mechanisms, real-time object detection and text recognition pipelines, and task-oriented interaction strategies adapted to the competition tasks. The system achieved reliable performance across most race tasks, although consistent results for the Forest task remained challenging. To investigate usability beyond the competition environment, we conducted a user study using simplified versions of selected race tasks. The study evaluated task completion time and success rate to assess how effectively the system could be used with minimal training. Results showed an average success rate of 93.46%, with reductions in task completion times for the evaluated tasks: Doorbell, Empty Seats, Grocery, Colours, and Touchscreen. This work serves as a technical report on the design, implementation, and evaluation of a Cybathon vision assistance system, providing insights into the integration of wearable hardware, multimodal feedback, and machine learning methods for assistive applications.

## Introduction

People with severe visual impairments face many challenges in daily life that sighted people do not encounter. Recent estimates indicate that globally more than 43 million people are blind and over 295 million live with moderate to severe visual impairments [1]. Everyday tasks such as finding objects on the floor or navigating through unfamiliar environments can become significant challenges that affect independence and overall quality of life, according to the World Health Organization [2].

Recent technological progress has led to a growing range of assistive solutions. Several recent reviews discuss new technology that visually impaired people can use to navigate daily life [3–6]. They highlight the differences between indoor and outdoor navigation. Indoors, localization can rely on dedicated tags or beacons [7–10], while outdoors, global navigation satellite systems are most widely used [11–13]. Computer vision-based systems combined with machine-learning methods for object detection [14–17] appear in both contexts and can help identify specific landmarks such as street crossings and objects. Traditional navigation tools, such as white canes, are being enhanced with electronic sensing devices using ultrasonic [18–22], radar [23–25], or LiDAR [26] sensors, which detect obstacles without physical contact and offer more detailed spatial information. Feedback mechanisms range from haptic cues through vibration or moving elements [18, 22, 27–29] to auditory feedback using spatial sound, special sound patterns or text-to-speech [4]. Beyond conveying text through Braille, assistive technologies have emerged that use various haptic displays such as shape and texture feedback in order to present graphic information from computers, tablets, and mobile phones [30–32].

Although some proposed solutions use standalone wearable computers or cloud-connected systems, many functionalities can be implemented directly on smartphones by leveraging their diverse sensors and feedback capabilities. Smartphones remain a more accessible and independent option for many users. This is supported by a study from 2022 in Ghana, where 46.9 % of 166 severely visually impaired or blind participants reported using a smartphone [33]. More recently, AI-based approaches using large language models have been proposed for real-time assistance [34–36].

Building on these technological advancements, a new Vision Assistance (VIS) race discipline for visually impaired participants was introduced at the Cybathlon in 2024. In this discipline, a visually impaired pilot aims at completing ten structured tasks [37, 38] within a set time limit using technology specifically developed for the challenge.

In this article, we present the technical approaches we applied to support a visually impaired pilot in completing the ten predefined VIS tasks. The VIS tasks represent specific, structured challenges inspired by daily living activities, providing a controlled competition environment to evaluate assistive technologies. While using assistive devices optimized for each individual task can improve performance for that specific task, it increases the total system’s size, weight and complexity. Therefore, we designed a system centered on a smartphone, and can handle the majority of tasks without requiring additional hardware. In addition, a smart rolling cane equipped with ultrasonic sensors and connected to the smartphone via Bluetooth was primarily used for obstacle avoidance tasks and, in other tasks, to provide accurate orientation information to move forward to the next task areas. All core functionalities are managed through a custom Android application running on the smartphone, which integrates computer vision, machine learning models, and auditory feedback via text-to-speech to guide the pilot through each task.

This work presents a technical approach to integrating existing smartphone capabilities with additional assistive devices to create a functional system tailored to the specific tasks of the competition. While recent advances in wearable technology, such as commercial smart glasses including Ray-Ban Meta glasses, provide highly lightweight and hands-free alternatives for assisting the visually impaired with daily objects [39] our smartphone-centric system focuses on balancing usability and competition-specific functionality without requiring extensive custom hardware. By prioritizing end-to-end task execution rather than isolated functionalities, our system demonstrates how current mobile devices can be adapted for the Cybathlon challenges. During the Cybathlon competition the proposed system was evaluated by a single, highly trained pilot. Therefore, to adequately assess the usability and user scalability of the system, and to systematically examine whether the smart vision assistance system can be effectively used by diverse users without intensive prior training, we present an additional user study and the results.

## Smart vision assistance system

The goal of developing the system was not only to create an assistive device capable of effectively performing the VIS tasks but also to enable users to complete the majority of these tasks using a smartphone that is convenient to carry. The system is built around a smartphone and is complemented by minimal task-specific attachments and a unified custom mobile application. This design allows all competition tasks to be performed within an integrated, consistent system.

### Hardware

The hardware of the Smart Vision Assistance system includes a smartphone, which handles the camera sensor and main processing, along with smartphone accessories such as a customized phone case, a mounting interface, a light funnel for the camera, and a stand. Additionally, the system features a wristband and a rolling cane that assist with obstacle detection and provide guidance for proper navigation orientation.

The primary requirements of the smartphone are a high-quality camera and sufficient processing power. In this study, a Google Pixel 7 Pro running Android 14 was used, as it is equipped with a high-quality camera and a powerful processor capable of executing machine learning models locally within an acceptable inference time.

**Fig. 1:**
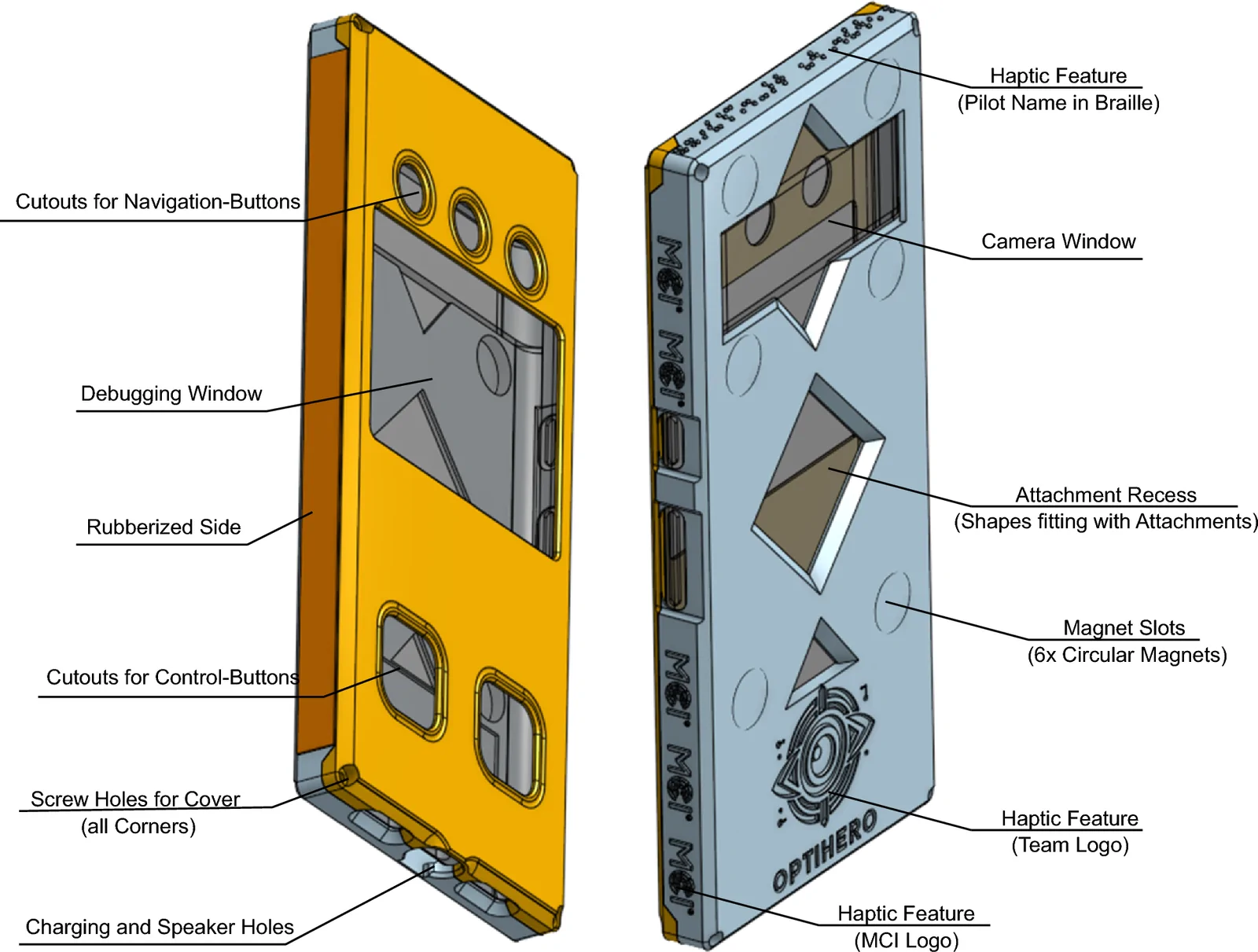
The 3D-printed phone case incorporates several functional design features. The case is composed of two halves that fasten together around the phone using screws. Haptic markers along the sides enable the pilot to quickly determine the phone’s orientation by touch. On the front, strategically placed cutouts align with the application’s UI elements to aid orientation and prevent unintentional screen interactions. The back features a pattern of cutouts and directionally aligned magnets that allow accessories to attach securely in predefined orientations

To improve handling, a custom 3D-printed phone case, shown in Fig. 1, was designed. It includes textured landmarks for the pilot to detect orientation, a rubberized side for better friction while being mounted for the Dish-Up and the Doorbell task and a custom magnetic mounting interface for various accessories.

Since our pilot had never used a touchscreen phone and was accustomed to a phone with physical haptic buttons, he initially experienced difficulties adapting to it. To address this, a screen cover was added. It is specifically designed for our app, featuring haptic landmarks and cutouts corresponding to the touchscreen areas intended for use within the app. To allow the pilot to use the camera while keeping their hands free, a magnetic mounting interface for the smartphone was developed. This mounting interface was strapped to the pilot’s chest using a belt system made of velcro. It allowed the phone to swivel between a downwards looking perspective to a horizontally looking perspective where it kept its position by relying on a screw that was tightened as the phone rotated up.

**Fig. 2:**
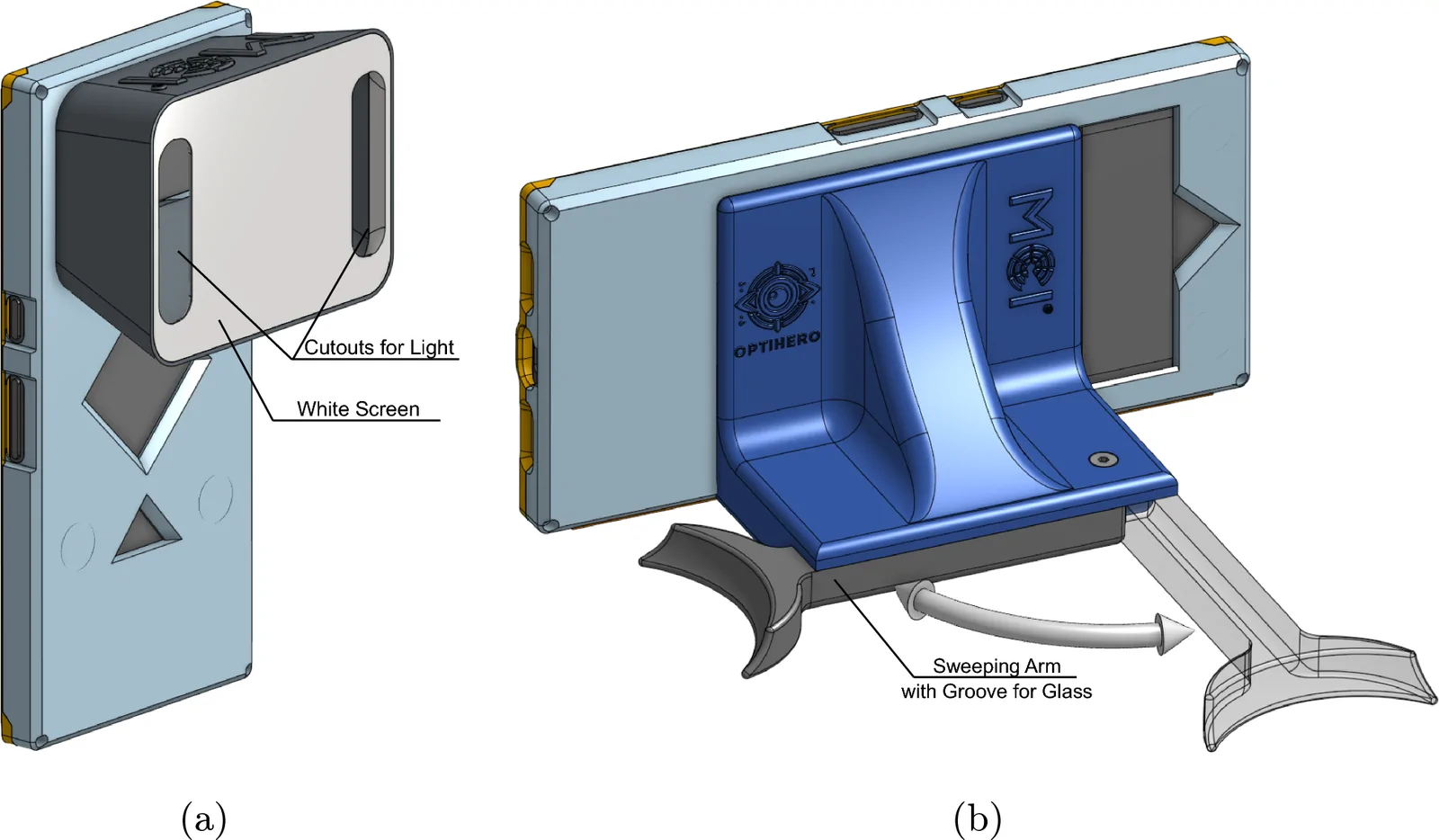
Additionally to the 3D-printed phone case, accessories are used to support certain tasks. In (a) the phone is shown with the magnetically attached funnel that restricts the light to the cameras for the Colours tasks to mitigate ambient illumination differences. In (b) a 3D-printed swingarm is used to keep a constant distance of the phone to objects in the Dish-Up and Doorbell task.

The cane is used to mount two additional accessories used together with the smartphone. One is a funnel with defined cutouts, shown in Fig. 2a, designed to restrict the camera’s field of view and to reduce ambient light, allowing illumination only through the cutout areas for the Colours task. Another accessory for the phone is a stand (see Fig. 2b), designed specifically for the Dish Up and Doorbell tasks. It includes a swiveling arm contacting the glass used in the Dish-Up task, while the phone, attached via its magnetic mount, faces slightly upward. This setup ensures a consistent distance and framing of the glass and allows the phone to provide reliable feedback. The same stand is also used for the Doorbell task, where the swiveling arm helps the pilot hold the phone at a consistent distance from the doorbell while scanning for the correct name. The magnets in the stand are oriented so it can only be attached in one orientation.

**Fig. 3:**
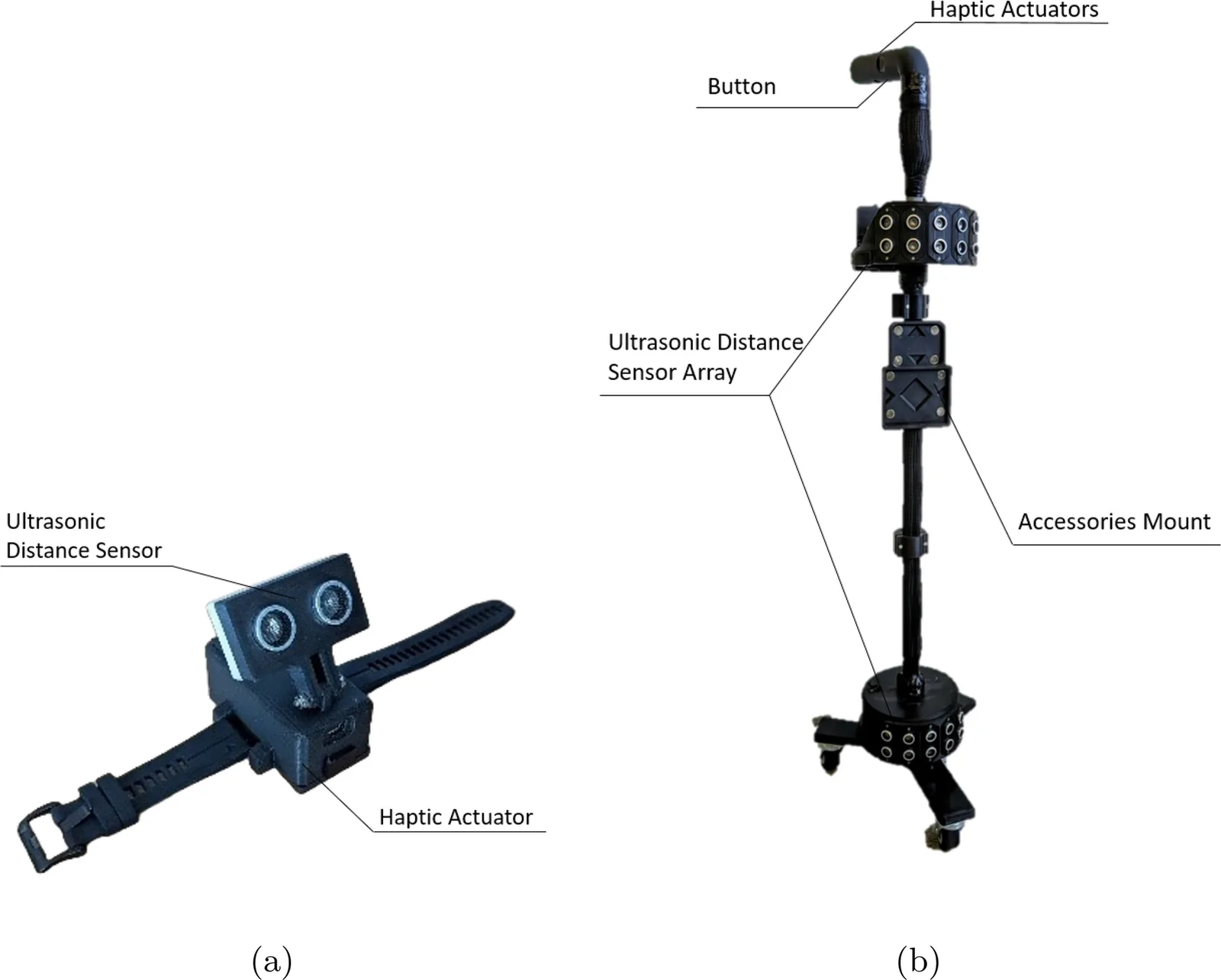
**a** The wristband is mounted on one of the forearms of the pilot to point to a specific direction in order to search for objects. **b** The rolling cane covers a much greater area than the wrist band. Furthermore, it can be used to recalibrate the pilot’s orientation in regards to the competition track and mounts are added to store the phone accessories when they are not needed

In several VIS tasks, the pilot must navigate the environment and estimate the distances to nearby objects. To support this, a rolling cane was developed to convey the surrounding information through additional sensory feedback. The cane, shown in Fig. 3b, contains two similar sensor modules, each with five ultrasonic sensors covering a $90^\circ$ field of view. Each module includes a microcontroller, a battery, a switch, and a status LED, and the modules are synchronized via a serial connection. The lower module communicates with the smartphone via Bluetooth and the upper module includes a servo motor that can tilt its sensor array up and down.

The handle contains three vibration motors that provide haptic feedback following the distance-based haptic rendering approach described by Kim et al. [29]. Additionally, the handle includes a button that the pilot can use to perform further actions. The rolling cane transmits the latest sensor values and the button state to the Android application, where they are interpreted to generate additional feedback. When a button press is registered, the smartphone application activates its reorientation feature as described in section “Navigation”. The cane also includes a carrying strap and magnetic mounting points for the smartphone accessories that are used in several tasks.

The wristband shown in Fig. 3a is a wearable sensing device designed to detect elevated obstacles during navigation tasks, specifically used in the Forest task. It is equipped with an ultrasonic distance sensor mounted on top of the enclosure and oriented forward to scan the space in front of the pilot’s upper body and head. Internally, the wristband contains an ESP32 NodeMCU microcontroller. The device is also equipped with a vibration motor integrated into the housing to provide haptic feedback. When the ultrasonic sensor in the wristband detects an object within a range of 20 to 100 cm, the ESP32 activates the vibration motor. This provides the pilot with an immediate alert to a nearby elevated obstacle, indicating the need for corrective action. The use of vibration feedback enables silent notifications that do not interfere with the audio cues provided by the cane.

### Software

#### Smartphone application

**Fig. 4:**
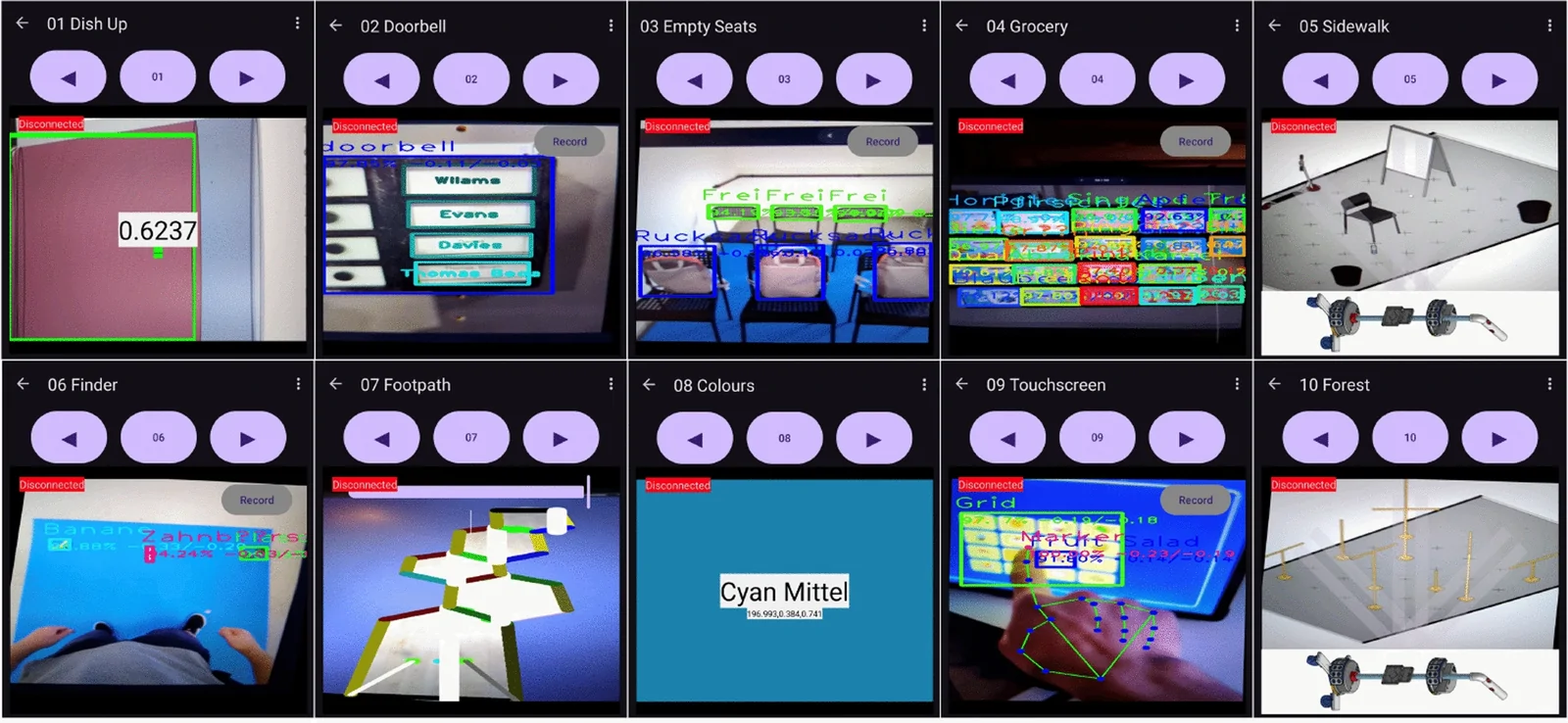
Each task has its own fragment in the smartphone application

The smartphone application, developed with the Android SDK in Java, is used for all tasks. It is the only interface through which the pilot interacts with the phone. The app consists of a single activity with multiple fragments, one for each task. To keep the implementation simple, each fragment maintains only the minimal state required during its use and resets this state whenever it is opened, ensuring that every task always starts from a clean setup. The fragments for each task are shown in Fig. 4.

**Fig. 5:**
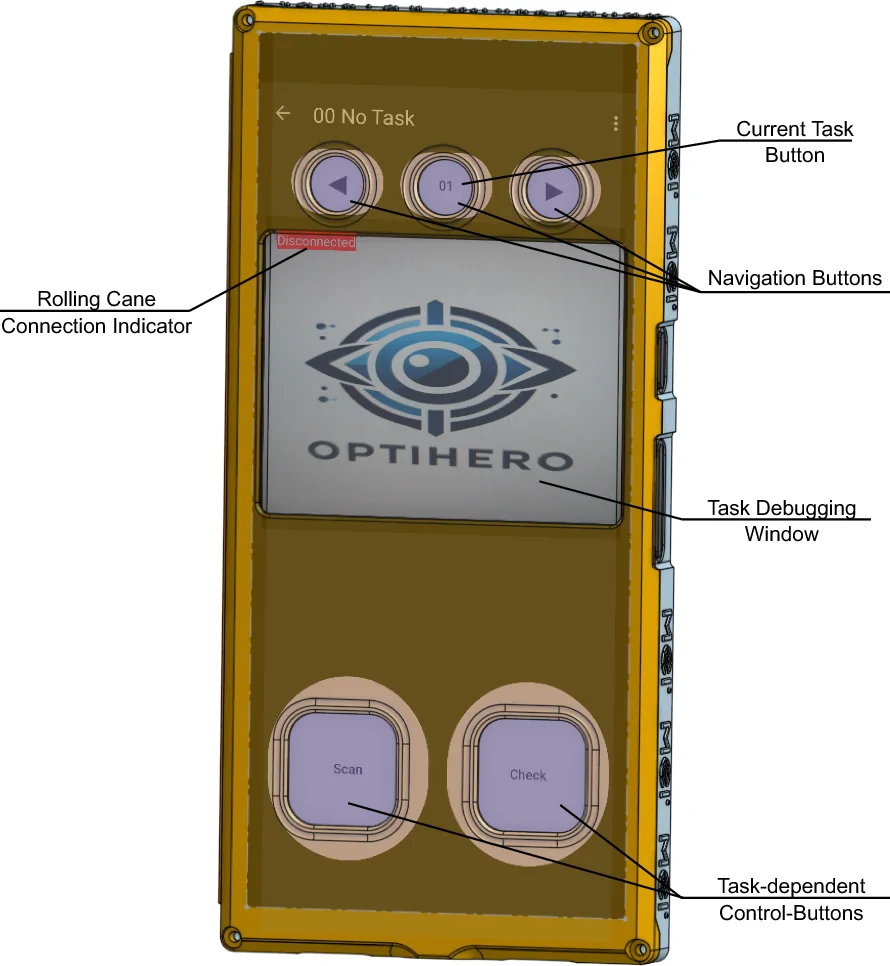
Via the upper buttons the fragments are navigated, whereas the middle and one of the arrows need to be touched at the same time to move to another task. The buttons on the bottom have a specific function related to the task. The rest of the screen is covered by the cover

One of the goals was to keep the app simple and consistent across all tasks. Therefore, a unified UI was developed for all tasks, providing consistency for our pilot, as shown in Fig. 5. The UI includes a row of three navigation buttons at the top that is shared by every task. The center button displays the current task number and can be pressed to show a short description of the task. Switching between tasks requires holding the center button together with either the left button or the right button for one second. This interaction was chosen to avoid unintended task changes.

To support the pilot in remembering the required steps, each task includes a description that is spoken using text-to-speech. All actions performed by the app are communicated through text-to-speech as well. For navigational feedback, the app also uses beeps that vary in frequency, volume, duration, and stereo channel. Bluetooth headphones are used so the pilot can clearly hear all audio cues. The beeping signals are also used to support navigation, as described in section “Navigation”. The app also provides two large buttons at the bottom, assigned specifically to the current task. If a task requires only one button, both buttons activate the same action so that the interactive areas of the UI remain in consistent physical positions. Whenever an action is successfully triggered, the pilot receives haptic feedback through vibration.

Almost all tasks in the app rely on computer vision and are implemented using the phone’s cameras. The app displays a large rectangular view showing the live camera image, primarily intended for debugging and troubleshooting during development. This view allows developers to see what the app detects and understand why certain actions succeed or fail during development.

Most of the image processing is implemented using the Java bindings for OpenCV. For tasks requiring object detection with custom models, YOLOv10-nano was used and trained in PyTorch on a PC. These models are lightweight enough to run efficiently on the phone while still providing reliable detection performance, as described in section “Model training”. The trained models are exported to ONNX and loaded onto the phone using the ONNX Runtime library.

**Fig. 6:**
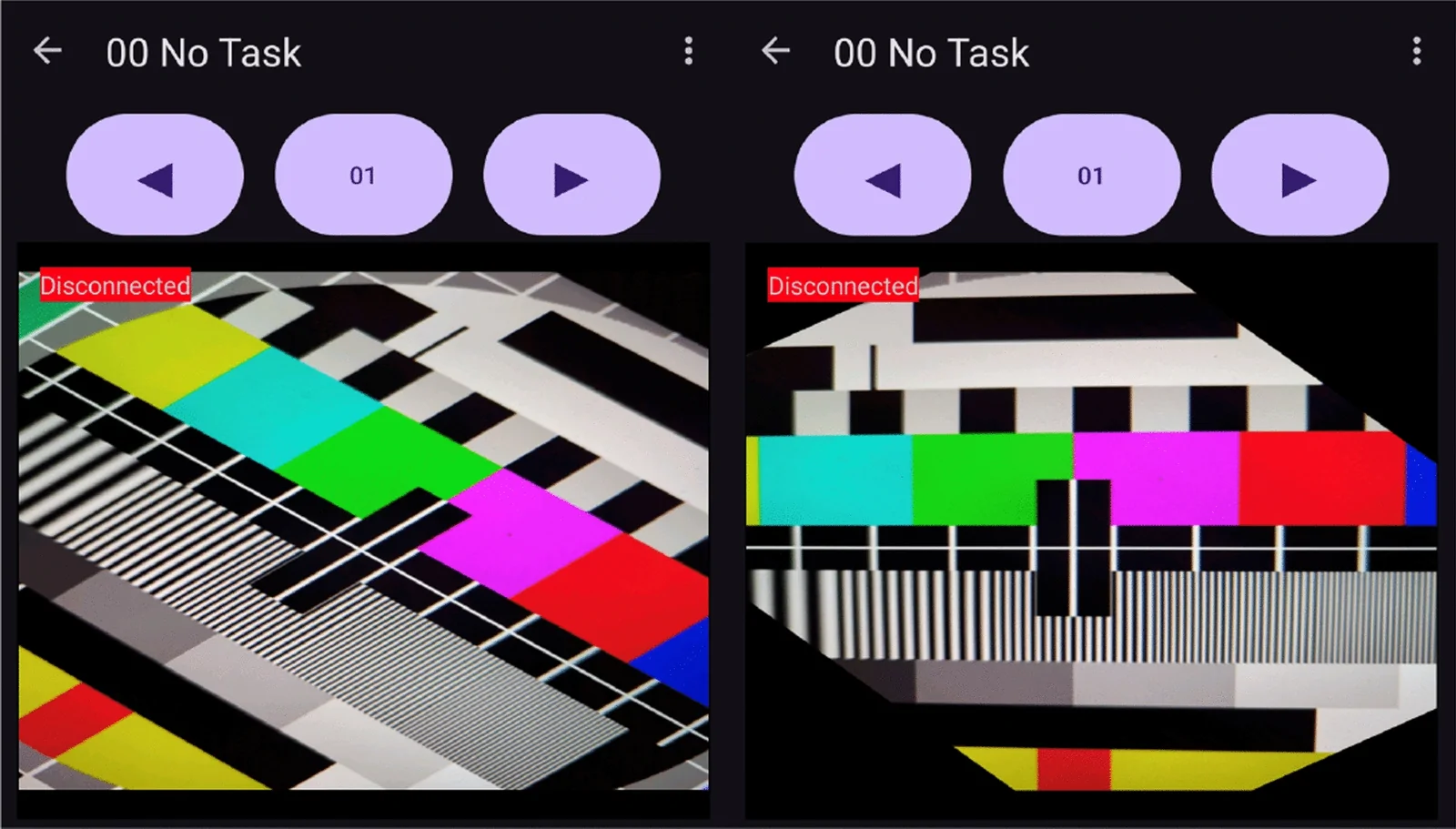
Image processing in app with auto rotation adjustment enabled (right) and disabled (left) that rotates images so they have the correct vertical and horizontal alignment, even if the phone is tilted

The app uses the phone’s orientation and acceleration sensors for several tasks. It primarily relies on Android’s game orientation mode, which ignores the magnetometer. This proved to be more stable over short periods, as the magnetometer is affected by the magnets in the phone case and tends to drift over time. In the dish-up task, the sensor data is used to detect whether the phone is resting on its side inside the stand. In the Grocery and Doorbell tasks, the app distinguishes between a downward-facing orientation for scanning and a horizontal orientation for searching. In the Footpath task, orientation changes are translated into near-instant auditory feedback that guides the pilot toward the walkway direction. The same directional feedback can be used in other tasks if the phone is mounted in a fixed position on the pilot’s chest and a button is pressed on the rolling cane. Across multiple tasks, the orientation data is also used to correct image rotation, ensuring that every captured frame is treated as if the phone were held horizontally. An example of a frame captured with and without automatic rotation is shown in Fig. 6. This makes image processing based on horizontal and vertical coordinates of the image more reliable and removes the need for our pilot to keep the phone oriented correctly along the camera plane.

The phone is connected to the rolling cane via Bluetooth. Through this connection, the app provides auditory feedback for the side-facing ultrasonic sensors that is used during the Sidewalk and Forest tasks, and it provides orientation-based feedback when the pilot presses a button on the cane handle.

#### Model training

The system relies on a mix of pre-trained and self-trained models. Pre-trained models include an OCR model for the Doorbell task and the Google MediaPipe hand-landmark model used to determine hand positions in the Touchscreen task. Self-trained models are used in the Doorbell task to detect column and text-field locations, in the empty-seat task to identify whether a chair is free or occupied, in the Grocery task to detect tea labels, in the Finder task to recognize objects on the floor, and in the Touchscreen task to detect the grid of squares and the target field. All models were trained on a computer in a Python environment using the Ultralytics YOLO library, with training executed on a GPU using PyTorch and CUDA.

**Fig. 7:**
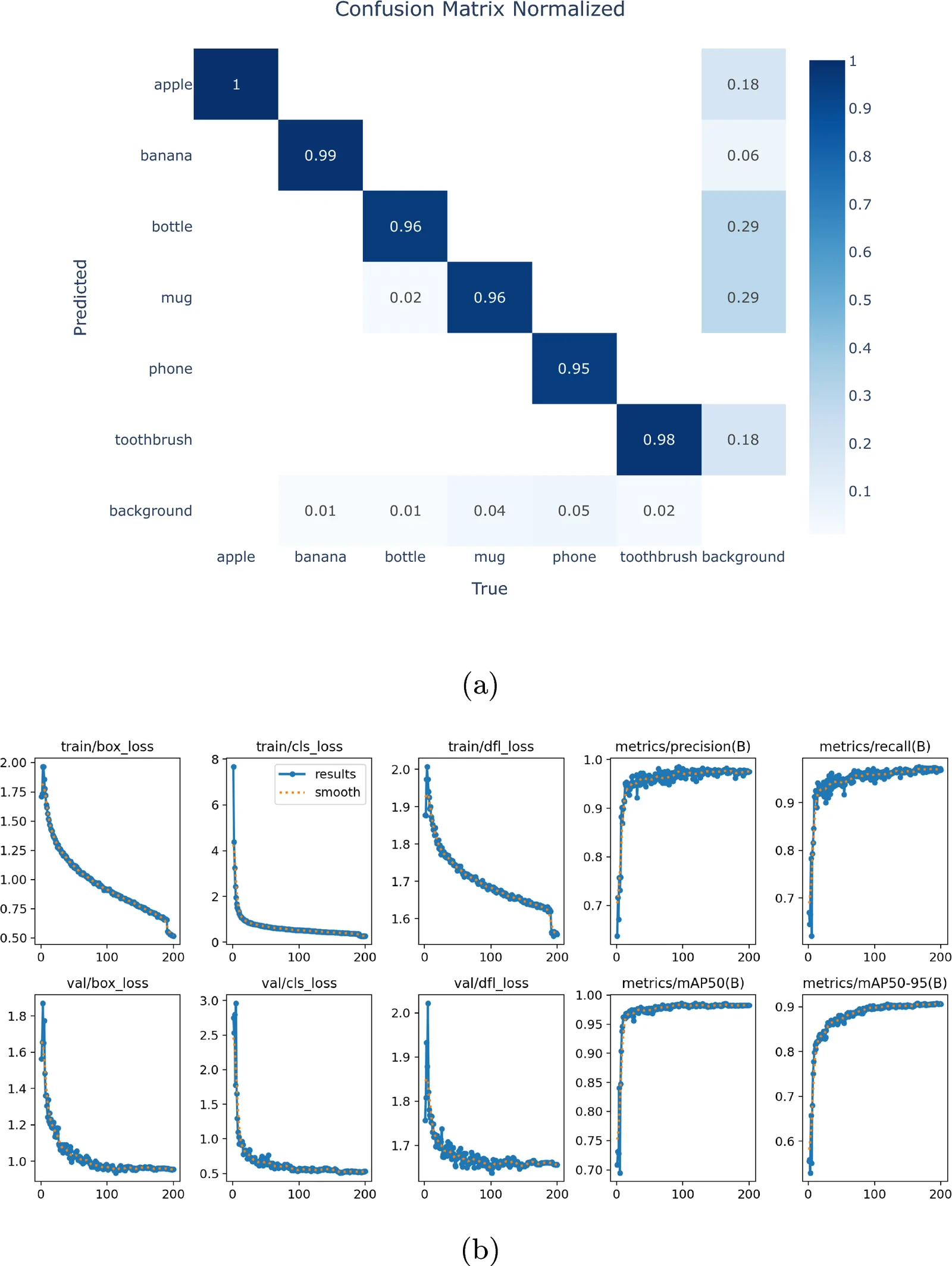
In **a** the confusion matrix from training the computer vision model for the Finder task is shown. It can be seen that the different objects are predicted with high accuracies and are distinguished well from the background

The task-specific datasets were managed using Roboflow, which allowed the team to share data and label images collaboratively. All dataset images were captured with the same device used during the tasks, the Google Pixel 7 Pro. Images were collected both by recording videos or taking photos with the phone’s camera app and by using the custom application to save frames with the same or slightly adjusted settings used during task execution. The training images were recorded for various angles and lighting conditions. For each new dataset, a small set of images was first uploaded to Roboflow through the web interface and labeled manually. Based on these annotations, Roboflow generated the dataset and applied augmentation to increase its size. The augmentation included random noise, rotations, brightness and colour adjustments, and occasional conversion to grayscale. The labeled datasets were then downloaded through the Roboflow API using the Roboflow Python library. A custom Python script combined multiple datasets into a single training set to further increase the number of training samples. Each model was trained for 200 epochs. After training, the resulting model was used to automatically label additional images. These automatically labeled images were uploaded back to Roboflow, where the annotations were manually checked and corrected using the web interface, extending and refining the dataset. The results of this training on the example of the Finder task are visualized in Fig. 7. This shows that there are still up to 5% of false positives for example for the phone. This is due to limited features that can be detected for some frames and due to a limited model size in favor of low latency. This was sufficient in the Cybathlon setting but may become limiting in less controlled and more cluttered environments, where more capable models may be required. Additional challenges for the model arise from low-light conditions and motion blur. To improve robustness against varying illumination, the training set includes images captured under different lighting conditions, including recordings taken directly on the Cybathlon course. In addition, the selected smartphone was chosen in part for its camera performance under low-light conditions. During pilot training, low-light environments proved to be one of the main sources of variability in detection performance. To reduce motion blur during operation, the pilot was instructed to move the phone slowly, while the application provides immediate feedback whenever an object is detected.

#### Navigation

For navigating through the course, the required distances were memorized by the pilot during training. To support this, the app repeats the step-by-step instructions for each task. Since maintaining orientation proved challenging, an additional feature was added. When the pilot presses a physical button on the handle of the rolling cane, the phone provides stereo auditory feedback based on the phone’s current orientation. The orientation is calibrated to the alignment of the Cybathlon track at the beginning of the race, allowing the pilot to realign when needed.

## Tasks

### Dish up

#### Task description

**Fig. 8:**
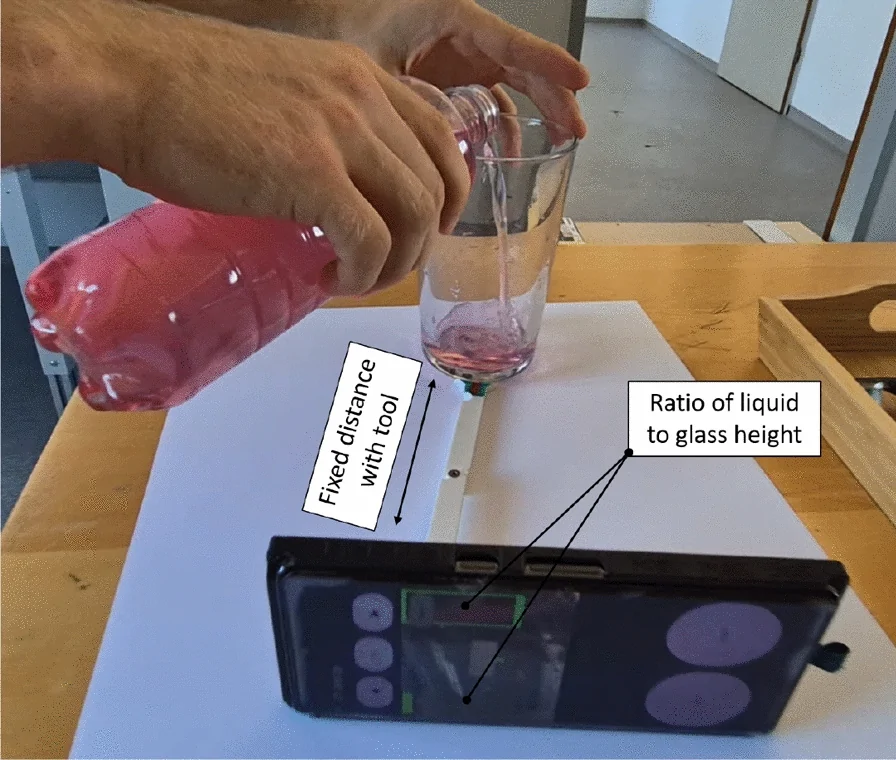
For filling up the glass, the phone is placed, using a distance tool that makes sure the glass is properly in frame. Audiofeedback is supplied dependent on the ratio between the glass and the filled liquid

In this task, the pilot must carry a plate with soup to a table and fill a glass with a bottle of red liquid. The glass must be filled to at least 2 cm below the rim, and the entire procedure must be completed without spilling or touching any liquid. An example of filling the glass is shown in Fig. 8.

#### Implementation

The application supports the pilot by providing auditory feedback that indicates how much liquid is in the glass and when to stop pouring. The app detects when the phone is resting on its side without movement, using the stand attachment as a positioning aid. The camera image is cropped so only the area containing the glass is processed. The liquid is detected using the L*a*b colour space by analyzing the third channel. The largest contour of the detected red area is selected as the liquid region. Once this region exceeds 17 % of the cropped frame and its lower edge is below 70 % of the screen height, the app starts emitting short, low-pitched beeps that rise in pitch as the liquid level increases. The rising pitch is intended to resemble the sound of a filling glass. When the glass is sufficiently filled and this is detected through the position of the upper contour edge, the beeps become longer and higher in pitch, indicating that the pilot should stop pouring.

#### Pilot workflow

The pilot begins with the rolling cane over his shoulder and the phone stand in his pocket. He navigates around the table with the tray, picks it up, turns 180°, and moves toward the table where the glass must be filled. He places the tray on the table, sets down the soup and spoon, attaches the phone stand, and presses the glass against the stand’s sweeping arm. He then opens the bottle and begins pouring slowly. As the liquid level rises, the app provides auditory feedback, with the pitch increasing accordingly. When the glass is filled, the beeps lengthen, signaling the pilot to stop. Finally, he closes the bottle, removes the phone stand, navigates around the table, and crosses the finish line.

### Doorbell

#### Task description

This task requires the pilot to identify and ring the correct doorbell on a multi-name door panel. At the start, the target name is printed on an A4 sheet placed on a table near the start line of the task. The arrangement of names on the panel is randomized between races, with all ten possible names present in each run: Smith, Jones, Williams, Brown, Davies, Wilson, Evans, Thomas, Robert, and Johnson. Each name tag on the panel may contain zero, one, two, or three names, representing different living arrangements such as couples, shared flats, or empty apartments. Only one of the names matches the target name, and pressing the wrong button results in task failure.

#### Implementation

The solution starts with identifying the target name using the phone’s main camera while it is held in the downward-facing position in the chest harness. OCR is used to read the text in the camera frames, and the extracted names are matched against a set of stored variants to improve reliability. For example, if the OCR reads "Janes", it is mapped to "Jones".

When the phone is tilted upward, it begins scanning for the doorbell panel and activates its automatic rotation correction. The panel and its individual labels are detected using a custom trained model with two classes, one for the full panel and one for each nametag. The model was trained as described in section “Model training” on a 3D-printed replica of the Cybathlon doorbell panel.

In the app, the OCR-detected names are assigned to the bounding boxes of the label detections. Using the bounding box of the full panel and any visible corner or edge, the application infers the location of each detected name within the grid. Each time labels are detected in the image, the smartphone emits a beep. If the correct name is visible, it uses a higher beep frequency. Once the correct label is detected in two consecutive frames, the app announces its position, for example "right side, second row from top".

#### Pilot workflow

**Fig. 9:**
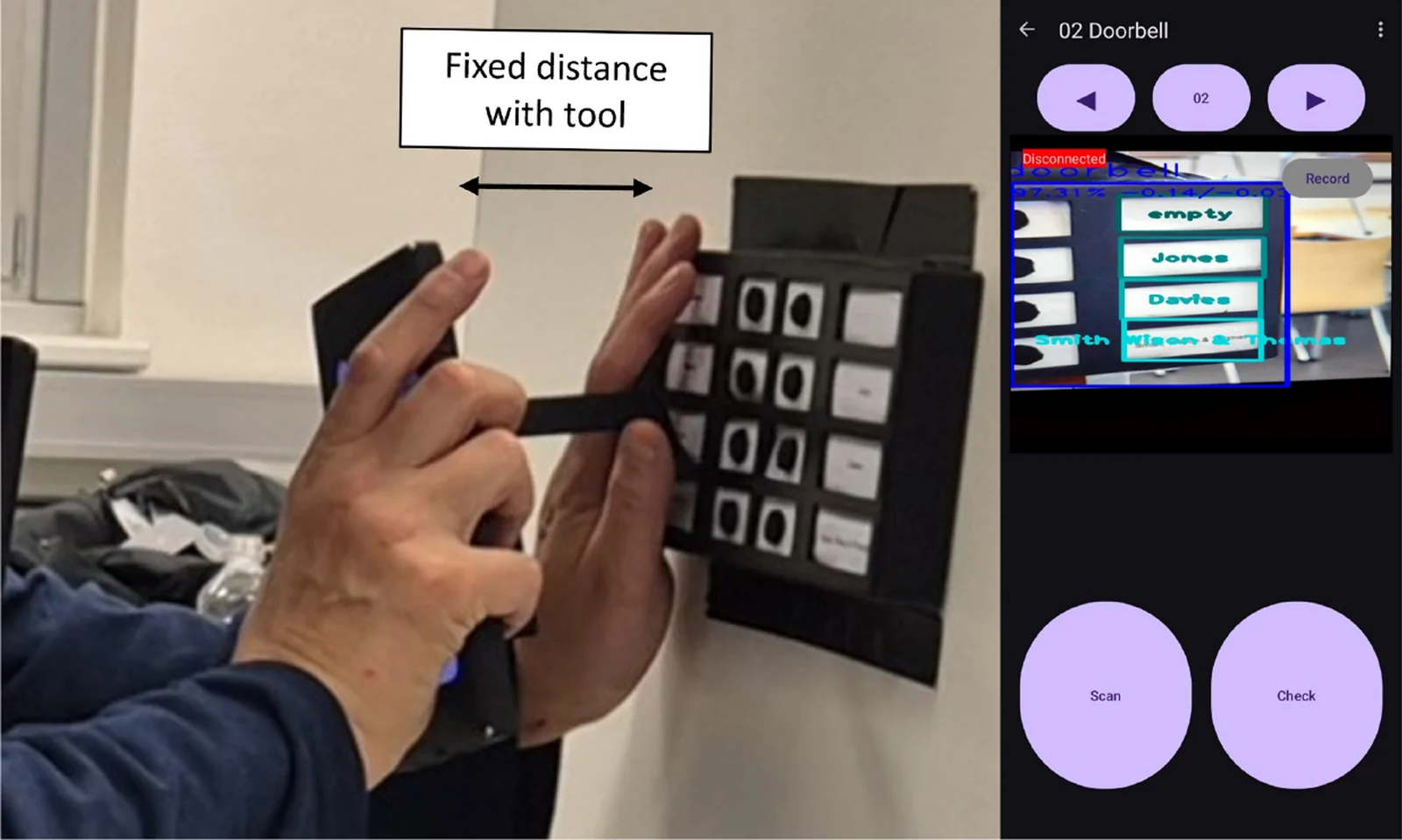
For scanning the signs in the Doorbell task, the same distance tool as in the Dish Up task was used. Going from left to right, audio feedback indicates to the pilot where to find the correct name to ring

The pilot begins by tilting the phone in the chest harness fully downward to enable scanning of the target label. Using the cane, he navigates to the box with the A4 sheet, stands in front of it, and scans it with the downward-facing camera. He then moves to the far side of the box to face the door, walks approximately two meters forward until the cane detects the doorframe or threshold, leaves the cane behind, stands on the threshold, and turns $90^\circ$ toward the doorbell panel. The phone is then removed from the chest strap and the stand accessory is attached magnetically. Using the arm of the stand to maintain the correct distance, the pilot slowly scans the panel, as shown in Fig. 9, pausing briefly to avoid motion blur. Once the target name is detected, the beep frequency increases and the app announces its position. The announcement can be repeated using either of the buttons in the smartphone application. The pilot presses the corresponding physical doorbell button, retrieves the cane, and passes through the doorway to complete the task.

### Empty seats

#### Task description

This task aims to challenge the ability to find a free seat in public places like buses or theaters independently and without unintentionally touching other people. The setup is composed of two rows of three seats, each of which can either be empty or occupied with a backpack or person, and a shelf with six cubes representing the chairs. The pilot must identify the empty seats without touching any objects, walk over to the shelf, and remove the cubes corresponding to empty seats.

#### Implementation

To solve this task, a self-trained object detection model was utilized, as described in section “Model training”. The model was trained with a dataset containing labeled images which show different scenarios of possible seat configurations, with the three classes ‘person’, ‘backpack’, and ‘chair’ represented by bounding boxes. For training the model, the exact chairs and backpacks specified by the Cybathlon guidelines [38] were used, and the ‘person’ label included a variety of people with different outfits, hairstyles, and stature. Training images were taken with the integrated recording feature of the app on different days with different lighting conditions and from different angles, to make the model more robust. To identify the chairs, the backrest was labeled, which is only visible when the seat is empty.

**Fig. 10:**
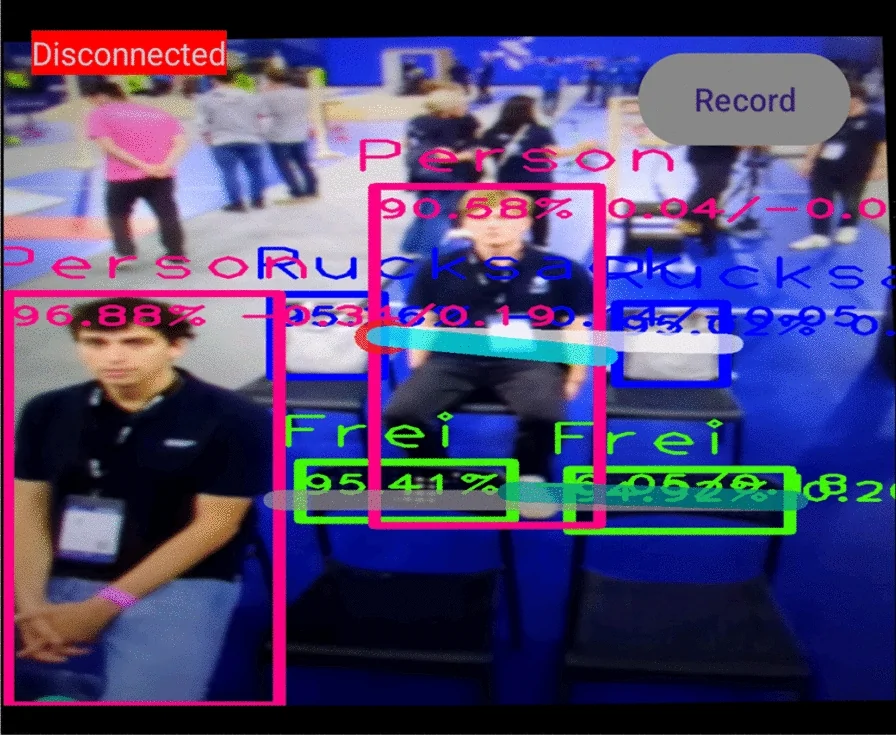
Visualization window showing the object detection and connection lines of the bounding boxes. Via audiofeedback the pilot is told, where a free seat can be found. In this configuration, the auditory feedback says "front middle and right empty, back nothing empty" in German languages (i.e."vorne Mitte und rechts frei, hinten nichts frei").

It was specified in the task definition [37] that there are always two people and at least one backpack in the scenario, and that there is at least one and maximum three empty seats. When the application is running, the algorithm counts the bounding boxes of the detected objects and checks whether these defined boundary conditions are met. Next, the center of the right edge of each bounding box is connected to the adjacent ones, to evaluate which combination results in two horizontal lines for the required 2x3 grid for the six seats. The algorithm therefore checks for the minimal distance between the center of the right box edges and the minimum angle of the connection lines to the horizontal. This is demonstrated in Fig. 10, which shows a section of the app interface for this task, with the detected bounding boxes, confidence thresholds, and connection lines visible in the visualization window. The detected configuration is stored in an array, and if a certain confidence threshold is reached in consecutive iterations, the pilot gets notified about the result acoustically.

#### Pilot workflow

The pilot uses the rolling cane to navigate towards the seats without touching any objects. When the acoustic signal of the cane indicates that the pilot is in front of the seats, he stops walking, removes the phone from the fixation in front of his chest, and holds the phone above his head with a slight downward inclination. He then makes a few side steps to the left and right, until the phone reads the detected configuration, which typically happens after a few seconds. The pilot puts the phone back into the fixture, takes the cane, and walks around the seats on the right side until he reaches the shelf. The detected configuration can be read out again at any time by pressing one of the two big buttons on the bottom of the phone screen. The pilot removes the cubes according to the results and passes the task.

### Grocery

#### Task description

The goal of this task is to help the pilot select the correct box from a shelf based solely on its label. The setup consists of a shelf with 20 identical cardboard boxes arranged in 4 rows and 5 columns. Each box carries a distinct tea label, and the exact labels are provided by Cybathlon. On a table in front of the shelf, one label is placed on a sheet of paper. The pilot must identify the corresponding box in the shelf, remove it without disturbing any other box, and place it on the table.

#### Implementation

A self-trained object detection model was created for this task, as described in section “Model training”. The model uses 20 classes, one for each label. The dataset includes the original digital labels, photos of printed labels, labels glued onto boxes, and labels placed inside shelves. Images were captured both with the phone’s default camera app and through the app’s built-in recording feature for setups that match the actual task.

**Fig. 11:**
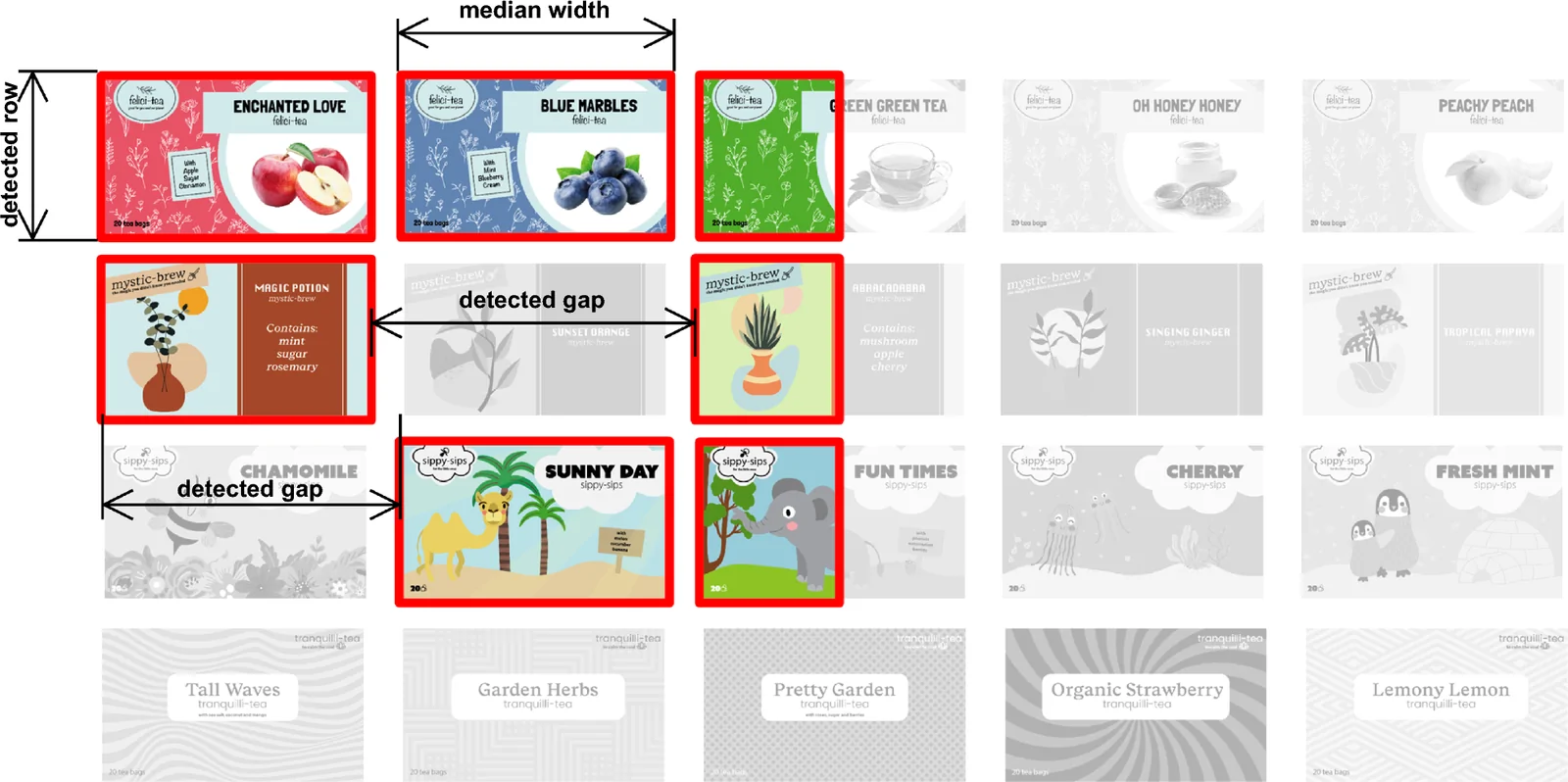
Grid visible to the camera and detections leading to only a part of the shelf per frame where gaps have to be filled

When the phone is tilted downward, a pitch below 40$^\circ$, the app assumes that the pilot is scanning the label on the table. In this mode, it disables orientation correction, switches to the main camera for higher image quality, and uses a confidence threshold of 50 %. For every frame, it selects the prediction with the highest confidence. If the same class is detected above threshold in two consecutive frames, the detected label is stored as the target, and the pilot is notified. Scanning can be repeated by long-pressing one of the interface buttons. When the phone is tilted upward, a pitch above 40$^\circ$, the confidence threshold is lowered to 10%, orientation correction is enabled, and the camera switches to the wide-angle lens to increase the visible portion of the shelf. If no label has been scanned yet, the app instructs the pilot to tilt the phone down again. If a label has already been scanned, the app repeats what it is looking for. During shelf scanning, every frame containing at least three distinct predictions triggers a short beep, indicating that labels have been detected. For each frame, the app builds a partial 2D grid based on the locations of the detected bounding boxes. Rows are formed by grouping boxes with similar vertical positions, and remaining boxes are processed until all detections are assigned to a row. Rows are then sorted vertically. Within each row, the boxes are sorted horizontally to assign a column order. Gaps are inserted whenever the spacing between boxes exceeds the median box width. Additional gaps are added at the beginning of a row if needed. The result is a partial grid representing the visible part of the shelf, as shown in Fig. 11.

**Fig. 12:**
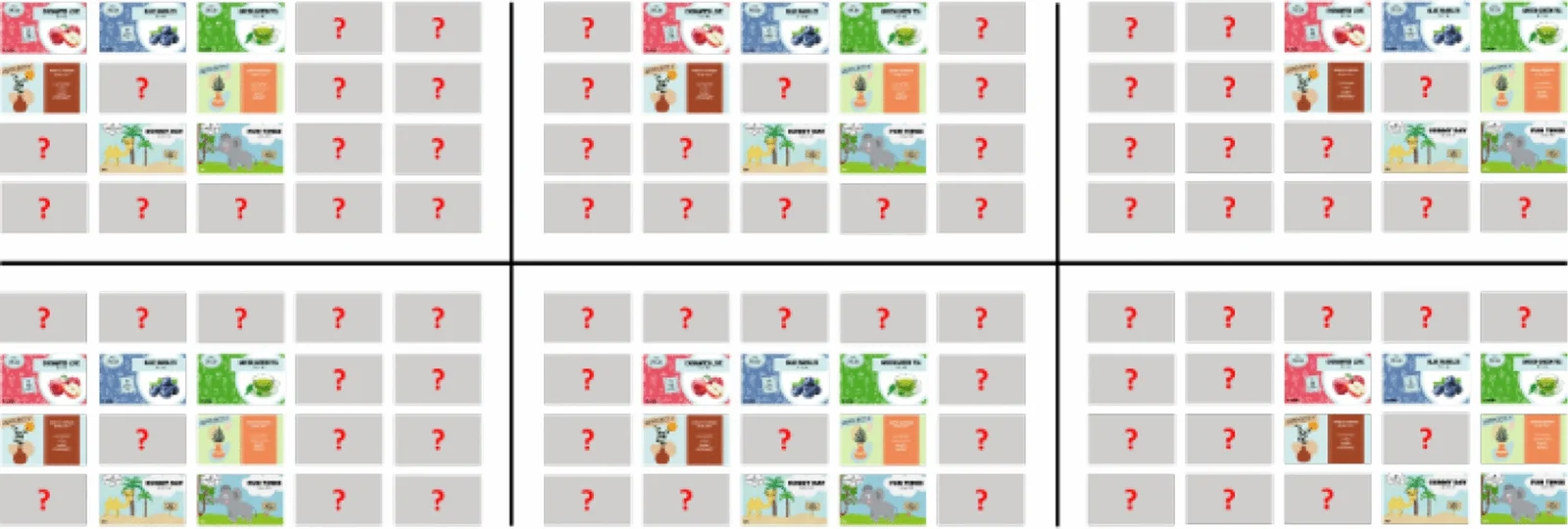
Possible locations for labels in the grid if only a part of it is visible. During scanning, the final position is set by assigning a weight and accumulating it

This partial grid is then matched against all possible placements within the full 4*× *5 layout. Each placement contributes a weight to the corresponding positions in the full matrix. Possible locations for a partial frame are shown in Fig. 12. Once all boxes have a unique position with the highest accumulated weight, the shelf is considered fully scanned. The app then tells the pilot the location of the target box using text to speech, describing its closest position from left or right and from the top or bottom. After this, the scanning beep increases in pitch. The pilot can repeat the description at any time by pressing one of the bottom buttons.

The approach assumes correct classifications, reasonably accurate label size estimates, and approximate horizontal and vertical alignment of the rows and columns which are supported by the automatic orientation based on camera tilt.

#### Pilot workflow

The pilot walks to the table using the cane’s feedback, positions himself in front of the label, and tilts the phone downward to start the label scan. Once the label is recognized, the app announces the target. The pilot then tilts the phone upward, turns around toward the shelf, and slowly moves left–right and up–down to scan all boxes. Every time at least three labels are visible, the app emits a beep, helping the pilot orient the phone. After the shelf has been fully scanned, the app informs the pilot of the target’s position, for example "second row from the top, second box from the right". The pitch of the scanning beep increases, and the pilot can repeat the position by pressing one of the interaction buttons. Finally, the pilot takes the correct box, places it on the table, walks around the shelf, and crosses the finish line.

### Sidewalk

#### Task description

In this task, the pilot must traverse a simulated sidewalk environment without colliding with any obstacles. The test area contains objects commonly found in urban environments, such as cylindrical barriers like trash bins, a chair, two scooters, an advertisement board and a small object on the ground representing a tripping hazard. This task evaluates the system’s ability to detect the environment and help the user navigate through the area safely. According to the competition rules, the pilot must not touch or manipulate any objects not intended for interaction.

#### Implementation

To address the challenges of the Sidewalk task, the rolling cane is used together with the mobile application. For reorienting after bypassing an obstacle, the task relies heavily on the reorientation feature activated by pressing the button on the cane handle, as described in section “Navigation”.

The Sidewalk task and the Forest task are the only tasks that do not use image processing. Instead, the phone is used to provide auditory feedback for obstacles detected by the side-facing ultrasonic sensors of the rolling cane, using stereo sound to indicate whether the obstacle is on the left or right. To reflect how close an obstacle is, the feedback scales with distance. Both the audio beep frequency and the vibration feedback increase as a stronger correction becomes necessary.

#### Pilot workflow

Once the pilot selects the Sidewalk task in the mobile application, the system enters its operating mode. Using the rolling cane, the pilot carefully scans the environment for nearby obstacles. Based on this input, the pilot receives feedback as detailed in Table 1. By responding to the vibrations from the cane and the audio cues from the phone, the pilot carefully navigates around the obstacles. Periodically, the pilot activates the reorientation feature to stay aligned after maneuvering around obstacles. After passing all obstacles, the pilot repositions and prepares for the next task.

**Table 1:** Overview of feedback signals for navigation assistance

Signal type	Source	Meaning	Pilot action
Audio beep (right)	Right headphone	Obstacle on the right	Steer left
Audio beep (left)	Left headphone	Obstacle on the left	Steer right
No beep	–	Clear path	Continue straight
Handle vibration	Cane handle	Obstacle ahead/path blocked	Stop immediately

### Finder

#### Task description

To complete this task, the pilot must identify one target item from six objects on the floor and find it without touching any of the other objects. The pilot must open a box to identify the target item and look for a similar object on the floor. The corresponding item must be picked up and placed in a bowl on the other side of the field to complete the task. The items of this task are a banana, a green apple, a yellow toothbrush, a green water bottle, a black mug and a black smartphone. They are placed on the floor in predefined positions in a random order, with three on each side when starting from the middle of the pitch.

#### Implementation

**Fig. 13:**
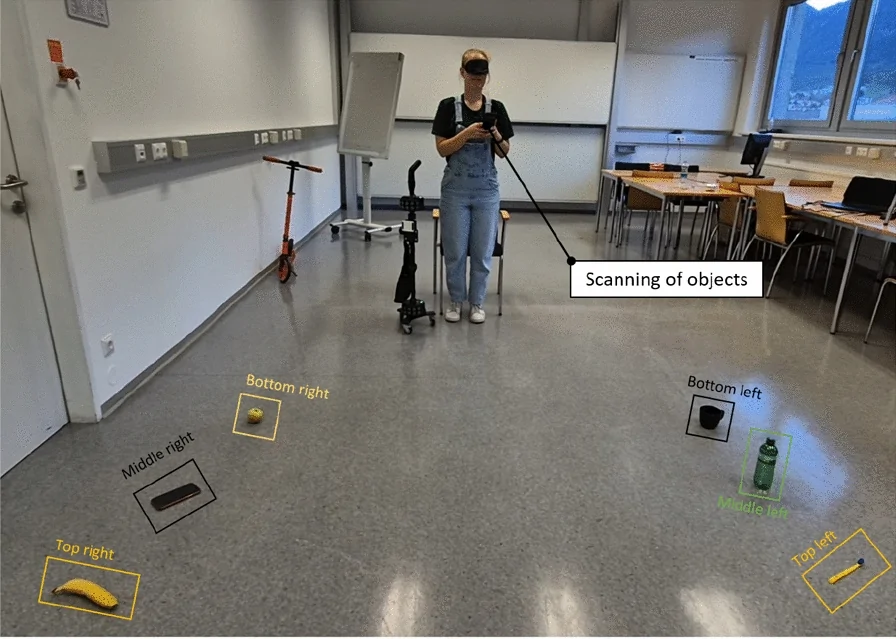
Real-time object detection with labels for the detected objects. The pilot is scanning left and right and gets audio feedback on the position of the recognized objects. After moving to the center, the objects are scanned again and taken

To solve this task, a model with six classes, corresponding to the six objects was trained, using the products specified in the task definition. The training dataset included both detailed, close-up images of individual objects from all different angles and perspectives, and images showing the whole scene with all six objects in one frame. The model was trained with the labeled images and tested, with the confidence level of the object detection displayed for each bounding box. Objects with less distinctive features, such as the phone, initially reached low detection confidence, so additional images of these classes were added to the dataset until detection became sufficiently robust. The phone was fixed in front of the pilot’s chest for this task, so when he was standing in the middle of the field and turned to the left or right, three out of the six objects were captured by the wide-angle camera feed and detected by the algorithm. If the algorithm finds three objects, the object names are read to the pilot starting from the left. The system recognizes whether the pilot is standing straight or is turned to the side compared to the calibrated starting position. Depending on the orientation, the item positions are either read out from top to bottom or from left to right. Figure 13 shows the real-time object detection of three objects on the left or right in real time, along with their class labels.

#### Pilot workflow

The pilot navigates towards the table containing the target item with the cane, opens the box and palpates the item with his hands and walks around the table and orientates himself to stand straight in front of the table in the middle of the field. While having the phone in the fixation in front of the chest, the pilot turns slowly to the left until the system reads out the three detected objects. If the target item was not part of it, the pilot turns to the right and repeats the procedure. When the pilot knows the target position, he walks five steps towards the middle of the field, leaves the cane in place, turns 90$^\circ$ to the corresponding side, and uses the target position confirmed by the application. They then take two small steps forward to pick up the item, which is in front, to the left, or right. After picking up the item, the pilot turns around in the direction of the cane, grabs it, and uses it to navigate to the bowl to place the item in it.

### Footpath

#### Task description

The Footpath task requires the pilot to walk from the start to the end of a wooden path without stepping off it. The path consists of five hexagonal plates of varying lengths. The first plate is always centered, and the direction of the following plates is randomized.

#### Implementation

The application uses OpenCV to detect the footpath and the pilot’s position in real time. To identify the wooden plates, the camera image is converted to the YCrCb colour space, which provides reliable separation of the wood from the surrounding floor under different lighting conditions. The pilot’s feet are marked with green stickers, allowing both feet to be detected, and the midpoint between them is taken as the pilot’s position in the frame.

**Fig. 14:**
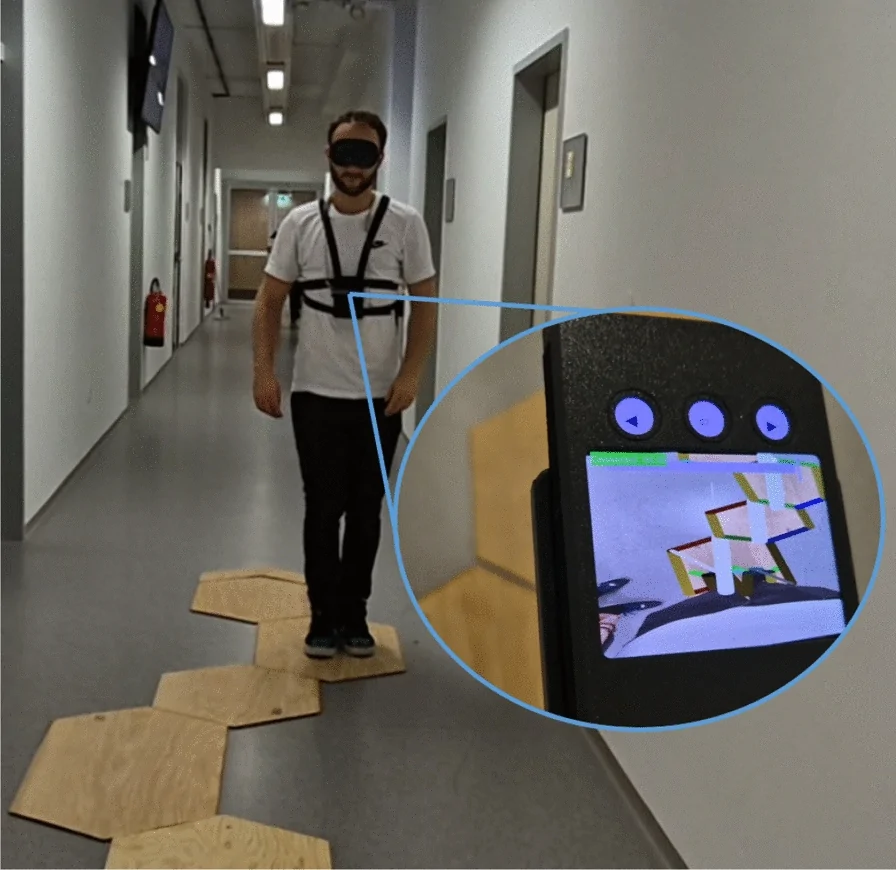
For the Footpath task, the phone is tilted about 90$^\circ$ towards the floor and scanning the tiles. Based on processing the edges of the tiles, feedback is given to the pilot to take a step forward or to the side

After detecting the feet, the app identifies the footpath. The wood-coloured areas are extracted, smoothed with a Gaussian blur, and processed with Canny edge detection. The resulting edges are dilated, and the largest contour closest to the pilot’s position is selected as the footpath. Small artifacts are removed with erosion, and the contour edges are approximated to obtain clearer lines. These lines are used to determine the rotation of the pilot relative to the direction of the path.

Based on this rotation, the original camera frame is rotated to align the path horizontally. The pilot’s position in the frame is recalculated accordingly. This alignment makes it easier to reconstruct the path’s layout and determine whether the pilot needs to reorient. The detected contour lines are used to identify the edges of each tile and derive the sequence of tiles ahead. An example of the detected tiles, as identified by the application, is shown in Fig. 14. The resulting information, the tile positions, the pilot’s location, and orientation, is used to generate continuous feedback.

#### Pilot workflow

The pilot begins by aligning with the start of the footpath, holding the phone facing downward. After taking the first step onto the path, the phone provides beeps to indicate if the pilot is rotated too far to the left or right. If the pilot drifts away from the center of the tile, the app notifies him that he is too far left or too far right. With small steps, the pilot moves forward. As he approaches the end of a tile, the app announces whether he should move left or right onto the next one. After stepping onto the new tile, the pilot reorients by following the beeping feedback and the positional cues. This process is repeated for all tiles until no further tile is detected. Once the app confirms that the path has ended, the pilot takes one final large step forward and crosses the finish line.

### Colours

#### Task description

This task requires sorting coloured fabric sheets on hangers based on their colour tone and brightness. Five predefined colour tones exist in three brightness levels, as specified by Cybathlon. Only specific tone pairs must be distinguished, including their brightness variations.

#### Implementation

For this task, the funnel phone attachment was developed to ensure consistent lighting for the camera. The camera image is transformed into the HSL colour space, and a nearest-neighbor classifier with manually defined weights is applied to identify the colours.

The light-funnel attachment, shown in Fig. 2a, blocks most ambient light and creates a controlled lighting chamber. The inner white surface helps the phone maintain stable white-balance calibration. All camera parameters, the focus, zoom, ISO, exposure, are fixed, and the camera flash is used as the sole light source.

To extract the colour from the image, the frame is cropped to the opening of the funnel. For stability, each frame is averaged with the previous frames. The average colour of this combined frame is then computed. If the new frame differs strongly from the previous one, the averaging is reset to handle quick colour changes. The resulting colour is compared to predefined reference values recorded from the actual fabrics. HSL was chosen because it separates hue and brightness clearly, matching the structure of the task. To match the colour, a weighted distance metric for hue, lightness, and saturation is used. Some colour tones are close to each other in this space, but because only specific tone pairs appear during the task, the app reports both possible colours in ambiguous cases and leaves the final decision to the pilot.

#### Pilot workflow

The pilot approaches the fabric sheets, attaches the light funnel, which can be attached either way, to the phone, and begins scanning. Each sheet is scanned by pressing it against the opening of the funnel and pressing one of the buttons on the phone. The app announces the detected colour, and the pilot places the hanger on the clothing rack accordingly. The pilot keeps track of the correct order by memory and uses the available rack space to position the fabrics. All sheets are scanned in this manner, and any previously placed sheet can be checked again at any time. Once the pilot believes the ordering is correct, he crosses to the finish line.

### Touchscreen

#### Task description

In this task, the pilot must press the correct icon on a touchscreen tablet. Cybathlon provides an Android application that displays a 4x4 grid of icons in a random position. The fruit salad icon is always located in one of the four central cells.

#### Implementation

A custom detection model was trained to identify the full grid, the fruit-salad icon within it, and a visual marker on the pilot’s finger. A small coloured marker on the index finger was used during the competition, as it was more reliable than Google MediaPipe hand-landmark detection, which was also tested but proved less stable when only a partial view of the hand was visible. Training images were recorded on different phones under varying lighting conditions, and the model was trained as described in section “Model training”.

**Fig. 15:**
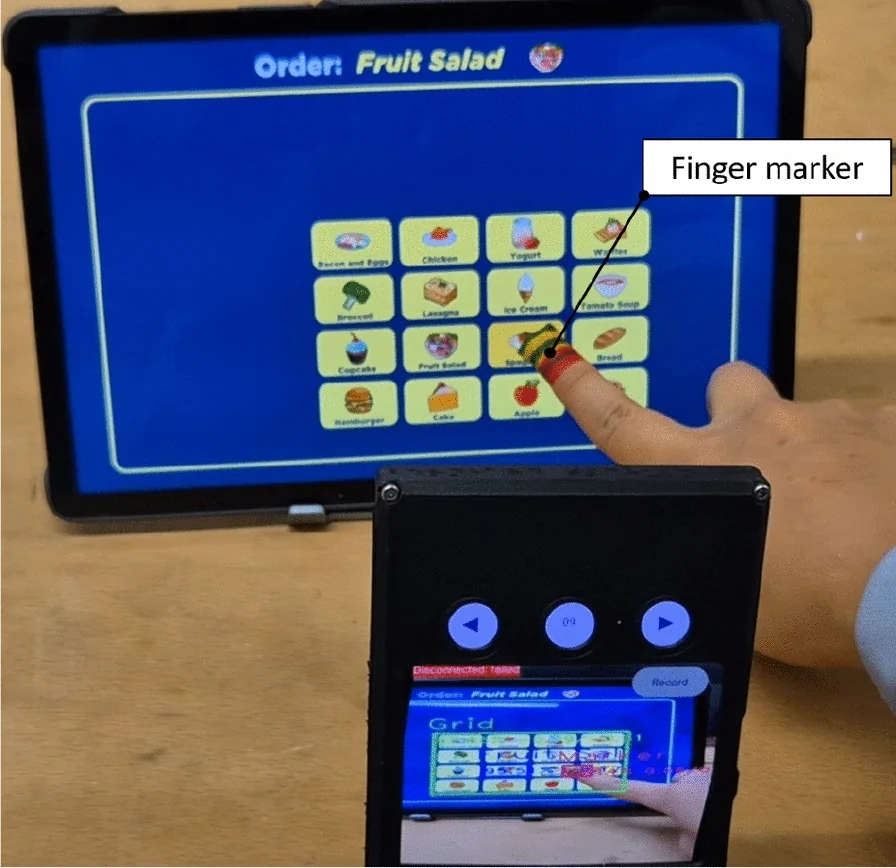
A coloured marker is used for the Touchscreen task to make sure the fingertip is robustly found by the computer vision algorithm. The hand is moved along the screen, based on audio feedback. Once the finger overlaps with the location of the fruit salad, the finger should press it

The app applies rotation compensation so that directional feedback (up/down/left/right) corresponds to the pilot’s orientation. The camera uses standard zoom and a very low confidence threshold (1%) to ensure that all relevant detections appear in mostly every frame even when the phone is moved and the image gets blurry. For each class, the bounding box with the highest score is selected. If MediaPipe landmarks are available, the marker detection is replaced with a small bounding box around the index-finger tip. An example showing how the grid, the fruit salad icon, and the marker on the finger are detected is provided in Fig. 15.

If the fruit-salad icon is temporarily occluded by the pilot’s hand, its last valid position is reused, as long as it was previously detected inside the grid. If the grid bounding box is fully visible, its scale is updated from the current frame. Otherwise, the last known scale is kept. Feedback is only given when all required classes are present (grid, fruit-salad icon, marker). The feedback direction is based on the relative position of the marker to the center of the fruit-salad icon. If the marker is aligned vertically, only left/right is announced; if aligned horizontally, only up/down is announced. When both match, the app reports that the finger is on the icon.

#### Pilot workflow

The pilot approaches the touchscreen using the rolling cane. Once positioned, he brings his hand to the tablet from the side until he touches the screen edge. With the phone in the chest strap pointing upward, both the display and the finger are visible to the camera. After an initial press to reveal the fruit-salad icon, the pilot keeps the finger on the screen while the app provides guidance.

The phone announces whether to move the finger up, down, left, or right. A higher beep indicates that the finger must move upward and a lower beep indicates downward movement; left and right are conveyed through audio channels with louder sound on the respective side. Spoken directions repeat every five seconds or whenever the required direction changes. Once the finger is positioned on the fruit-salad icon, the beeping changes in duration and the app confirms the position via speech. The pilot then lifts the finger and presses the icon. After completing the input, he navigates around the touchscreen and crosses the finish line.

### Forest

#### Task description

The pilot must navigate through a simulated environment that includes obstacles at various heights. These include both low positioned and elevated horizontal bars that simulate real-world hazards such as twigs from a tree. The goal is to traverse the space without making contact with any obstacles. This task evaluates the system’s capability to respond to obstacles at different vertical positions to prevent collisions.

#### Implementation

A solution concept similar to the Sidewalk task in section “Sidewalk”, was used to address the challenges of the forest task. The pilot uses the rolling cane in conjunction with the mobile application. The cane is equipped with ultrasonic sensors that continuously scan the lower environment for obstacles near foot level.

In addition to the cane, the pilot wears a wristband with an ultrasonic sensor positioned to detect elevated obstacles, such as bars at upper body or head height. This dual-sensor system enables the device to distinguish between low- and high-level hazards.

Similar to the Sidewalk task, the system provides auditory signals through the mobile app to guide the pilot in avoiding collisions. When the wrist sensor detects a high-level obstacle, the wristband vibrates to prompt the pilot to stop. For low-level obstacles detected by the cane, the system generates specific steering commands, same as described in section “Sidewalk”.

#### Pilot workflow

This task is done in a similar manner to the Sidewalk task. When the pilot switches to the Forest task, the system activates audio feedback whenever the side-facing sensors of the rolling cane detect an obstacle. The pilot walks forward carefully and scans the surroundings using both the rolling cane and, for this task in particular, the wristband to detect higher obstacles. Orientation is maintained in the same way as in the Sidewalk task, by pressing the button on the rolling cane to activate the reorientation feature described in section “Navigation”. Upon completing this final task, the pilot reaches the end of the course and finishes the race.

## User study

### Setup

To evaluate the implemented solutions for the Cybathlon, a user study was conducted. The study protocol was reviewed, approved by the University Ethics Committee, and received a positive assessment under reference number 20250709. A total of 11 participants took part in the study, comprising four female participants with an average age of 28 years and seven male participants with an average age of 27 years. All participants were healthy and reported no impairments related to vision or the musculoskeletal system. During the study, each participant wore blindfolds while completing the timed trials. The study included the Doorbell, Empty Seats, Grocery, Colours and Touchscreen tasks, executed in the order as named. While the original Cybathlon track is arranged in a long linear sequence within an arena, the number of tasks was reduced due to time limitations and available space. The tasks were also spatially rearranged to better fit the environment and to reduce the risk of collisions while participants were wearing a blindfold. As a result, no navigation-based tasks were included in the study.

The study was not designed to simulate the competition environment of the Cybathlon, but rather to explore whether the implemented solutions are suitable for use without extensive training. Hence, participants took a break in between the tasks, where the blindfold could be also removed. Before the tasks are timed, the participants can get used to the proposed devices for a period of five to ten minutes for each task with and without the blindfold. The task is started, if the participant communicated that they feel ready to be timed. Following the training period, ten trials were conducted per participant and task, except the Colours task. As the time of the Colours task was substantially longer than the others, only five trials per participant were completed. The data that was collected during each trial was completion time in seconds and outcome as success or failure according to the rules set by Cybathlon [37].

**Fig. 16:**
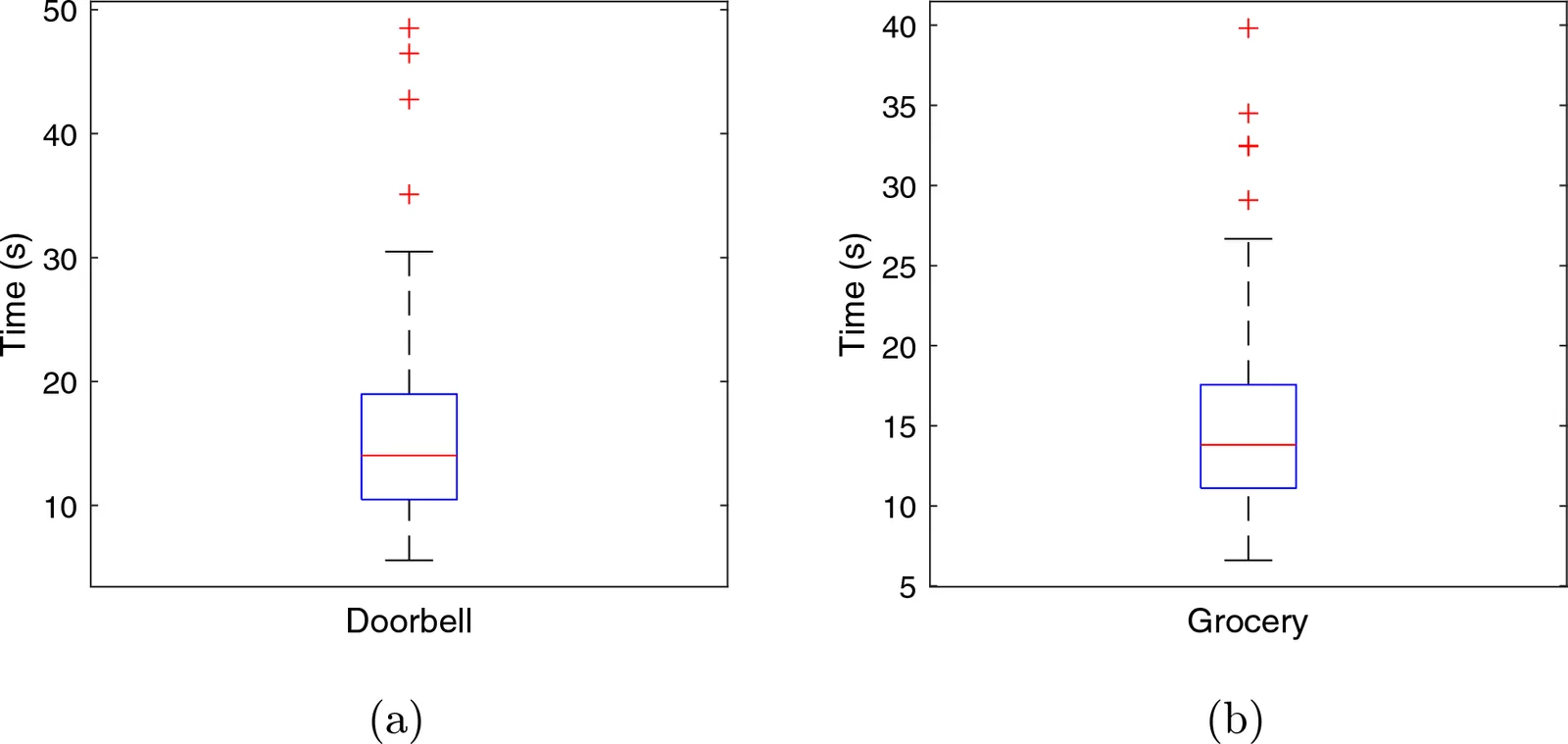
For the Doorbell task, a median completion time of 14 s was achieved, with non-outlier maximum of 30 s and a minimum of 6 s, whereas 50 % of all trials were between 10 s and 19 s. For the Grocery task, the median completion time was 14  s with non-outlier maximum of 27 s and a minimum of 6 s, while 50 % of all trials fell between 11 s and 18 s

### Results

**Table 2:** Mean time, standard deviation, coefficient of variation and error rate per task including all participants

Task	$\tilde{x}$ (s)	*σ * (s)	*CV*	*e* (%)
Doorbell	14	8	0.5	3.6
Empty seats	11	12	0.8	6.4
Grocery	14	6	0.4	3.6
Colours	70	29	0.4	3.6
Touchscreen	11	8	0.6	15.5

Based on the collected data, median time $\tilde{x}$, standard deviation *σ *, coefficient of variation *CV* and the error rate *e* were calculated per task respectively. The calculation of *CV* is based on Equation 1. All of the metrics are shown in Table 2. The metrics show, that the median time is between 10 and 15 s for each task, except the Colours task. Here, the median time with 70 s and also the standard deviation is much longer. However, in relation to the mean completion time of the task, the variation of the Colours task is the same as for the Grocery task and smaller than all others. Based on *CV* the Empty Seats task has shown the highest variation. In terms of successfully completed trials, *e* was lowest with four failures out of 110 trials for the Doorbell and the Grocery task and two failures out of 55 trials for the Colours task. It was highest for the Touchscreen task with 17 failures out of 110 trials.

1 $$\begin{aligned} CV = \frac{\sigma }{\overline{x}} \end{aligned}$$

**Fig. 17:**
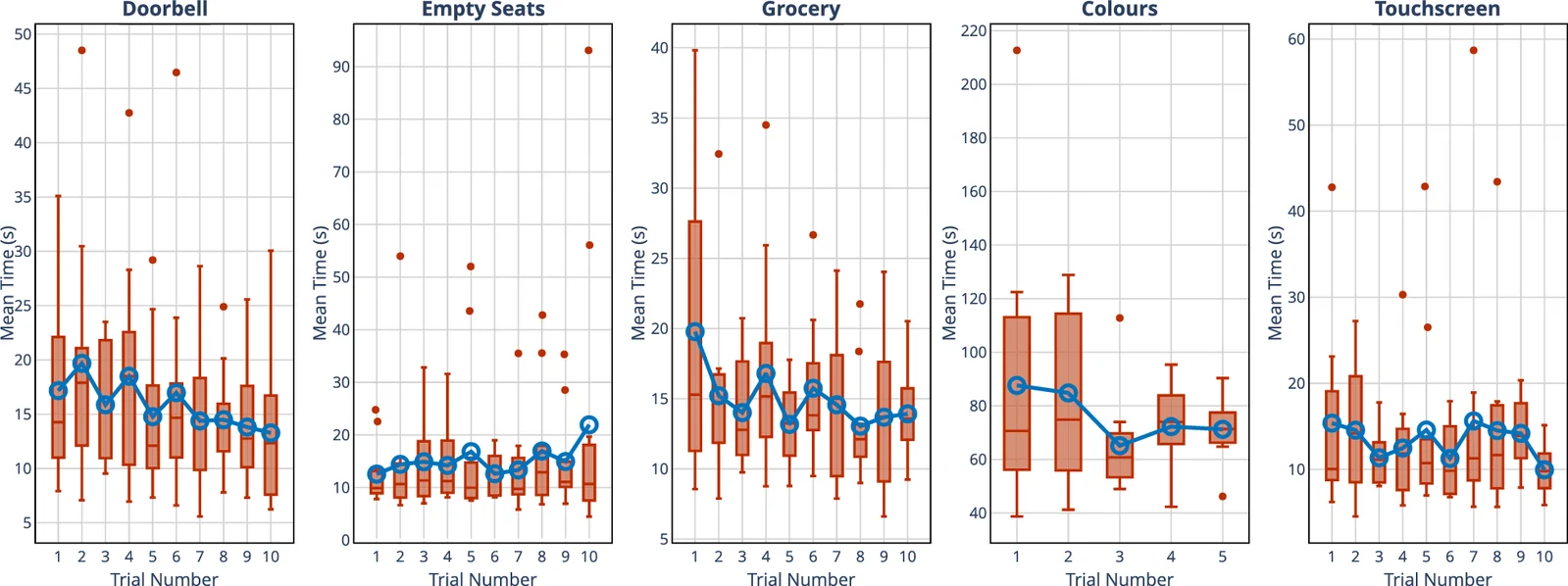
For all five tasks, the learning curve across the trials of all 11 participants is plotted against the mean time needed and boxplots

**Fig. 18:**
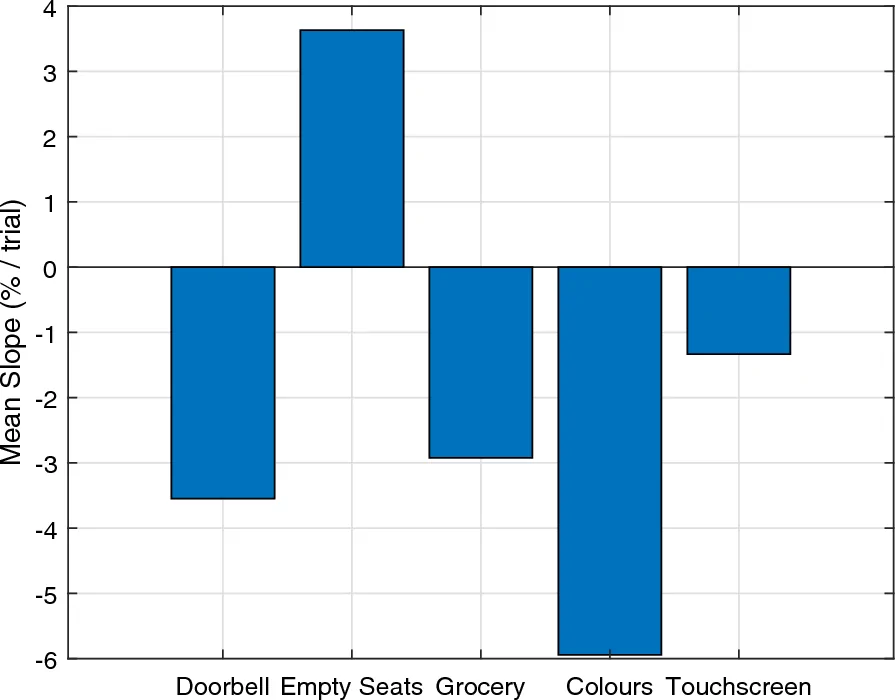
Based on the mean times across all participants as a function of trial number, an average slope was fitted using the least squares algorithm. This slope was then normalized by the overall mean trial time and plotted for all tasks

Figure 16a, b show the results for the Doorbell and the Grocery tasks in more detail. It can be seen that outliers occur primarily for longer task durations, whereas 50 % of the data lies within the 10 to 19 s and 11 to 18 s respectively. For other tasks, outlier also appear only for very long completion times. To understand the learning effect and consistency of using the technology, the mean times of all participants are plotted across the number of trials for each task in Fig. 17. Based on the data, a linear function is fitted using the least squares method to find the slope of the data. If the slope is positive, it indicates that the time required to complete the task increases with the number of trials and vice versa. The slope is normalized with respect to the overall mean completion time and shown in Fig. 18.

## Discussion

In the case study, the smartphone based Doorbell, Empty Seats, Grocery, Colours and Touchscreen tasks are evaluated. Overall, the error rates are low, especially if it is considered that the training time with the devices was as short as a few minutes per task. The only task where the error rate was increased was the Touchscreen task. During the conduction of the study, it was observed that for this task it was challenging to find the right amount of feedback that works for a specific person. The current setup provides feedback tailored to a competition scenario where speed is critical. Most visually impaired smartphone users are accustomed to fast speech rates when using screen readers such as VoiceOver or TalkBack. However, for sighted subjects who are not familiar with screen readers, this may have posed a greater challenge. However, for some participants, the feedback was delivered too quickly, leading to most errors occurring when they attempted to press the target button on the touchscreen slightly too early. If the application were to be explored for broader real-world deployment, personalizing the feedback methods would be expected to mitigate this issue. As the median time and standard deviation are acceptable, the implementation approach can be considered effective.

While for the Doorbell task the error rate was very low, the median time is slightly longer than for the Empty Seats and Touchscreen tasks. It is expected that this is due to the part of scanning the Doorbell sign. Here, the OCR model works fast and reliable, but maintaining the correct distance and orientation can be challenging. In the future, this could potentially be improved by adding scaling and perspective transformations before processing the written text. Another factor to consider is motion blur and that this task, as well as the Empty Seats task, requires more steps to be done and memorized compared to the Touchscreen task.

The data related to the Grocery task show that it works reliably, fast and with comparably low variability between trials. It was noticed that keeping the right distance and orientation to the scanned objects is easier, compared to the Empty Seats and Doorbell task. Similar to the Touchscreen task, it seems most intuitive to orient oneself facing the objects and around an arms length away. The higher reliability compared to the Touchscreen task is expected, as the scanned objects can be touched while searching for the target, which simplifies providing feedback.

The Colours task had the longest completion time; however, the variability between trials and the error rate were quite low. The long completion times are partly due to the complexity of the task, but also because the endpoint of the task is less determined. As the participants communicate when they are finished, they often sorted the Colours first but then re-checked all the hangers to ensure that they have been sorted correctly. This creates a trade-off between reliability and speed, which resulted in longer completion times, as in this study participants focused on correct execution rather than speed.

Learning effects of the participants across the study was investigated by looking at the completion times of the tasks per trial. It can be seen in Fig. 18, that four out of five tasks showed slight improvement with repetition. Only the Empty Seats task showed an increase in completion time. However, the reason for this is expected to be due to the phone repeatedly running a resource-heavy classification algorithm, creating heat and therefore slowing down over time. As the task was conducted only over a very short period of time in the competition, it was not as apparent as in the study. However, to scale the solution beyond the Cybathlon competition tasks, the structure of the algorithm would need to be optimized. The rate of improvement of the other four tasks is noticeable but in a small range. This indicates that it is intuitive to use already after a short period of training with the technology. Also, it should be noted, that this data includes also failed attempts. It is expected that with an extended amount of training sessions, the normalized mean slopes would get even closer to 0.

While the results of the study indicate that the system performs reliably for the evaluated competition tasks, the trained task-specific models are not intended to provide general-purpose assistance for visually impaired users in unrestricted real-world environments. Furthermore, to determine whether such a system provides a quantifiable benefit to visually impaired individuals in daily life, future comparative studies are necessary. Within the constraints and reliability requirements of the Cybathlon competition, the self-trained YOLO-based approach provided a robust and dependable solution for the defined tasks. However, we believe that recent approaches based on real-time vision-language models as well as generalized open-vocabulary detection models, could provide promising directions for broader assistive applications in daily living environments for visually impaired users.

## Conclusion

In this article, we present the technologies used for the VIS race during the Cybathlon 2024. While all of the technology used in the race is described in detail, a small user study was also conducted for the smartphone-based tasks of Doorbell, Empty Seats, Grocery, Colours, and Touchscreen. In the scenario of the competition, on average 48 s would have been available for each task. From the five evaluated tasks, the mean time would have been well within the limit with the exception of the Colours task. However, with transitions, reorientation and longer tasks, performing all the tasks within eight minutes is probably not feasible for our solutions yet.

The error rates demonstrate that the system is capable of performing the designated competition tasks under relaxed time constraints. The evaluation has shown that in the future, the focus should be on improving the feedback as well as achieving more efficient use of hardware resources. A limitation of the study is that it was limited to single tasks, rather than linking them together as well as the fact that participants of the study were only wearing a blindfold. This makes it difficult to compare with individuals with congenital blindness as well as to evaluate them against other assistive devices.

Regarding the design of the tasks, particularly based on the pilot’s experience, it was suggested that the Colours and Touchscreen tasks could be improved to make them more applicable for everyday use. If a touchscreen is used for public use, it should be easy enough to make it accessible to the visually impaired by design, rather than forcing the use of additional technology.

Overall, the Cybathlon 2024 was a big driver to come up with new assistive technologies for the vision impaired. The solutions presented in this article offer cost-effective assistance and portability especially for the smartphone based tasks that were evaluated in the user study. Furthermore, the findings from this study suggest that even short training periods are sufficient for users to become familiar with the technology, highlighting its intuitiveness and ease of adoption. However, the variability in the study also shows that the performance during tasks still strongly depends on individual familiarity with the system and the specific interaction conditions during use. Future work should focus on integrating multiple tasks into a continuous workflow, personalizing feedback for individual users, and optimizing both software and hardware performance. By addressing these aspects, the presented solutions offer practical insights into the integration of multimodal feedback and machine learning methods, serving as a technical foundation for future developments in assistive technology.

## Data Availability

The datasets generated will be available from the authors upon request.
